# How are podocytes affected in nail–patella syndrome?

**DOI:** 10.1007/s00467-007-0714-9

**Published:** 2008-07-01

**Authors:** Ralph Witzgall

**Affiliations:** University of Regensburg, Institute for Molecular and Cellular Anatomy, Universitätsstrasse 31, 93053 Regensburg, Germany

**Keywords:** LMX1B, Mouse model, Podocyte foot process, Slit diaphragm, Promotor analysis, Target genes

## Abstract

Nail–patella syndrome is an autosomal-dominant hereditary disease named for dysplastic fingernails and toenails and hypoplastic or absent kneecaps evident in patients with the syndrome. Prognosis is determined by the nephropathy that develops in many such patients. Besides podocyte foot-process effacement, pathognomonic changes in the kidney comprise electron-lucent areas and fibrillar inclusions in the glomerular basement membrane. These characteristic symptoms are caused by mutations in the gene encoding the transcription factor *LMX1B*, a member of the LIM-homeodomain gene family. Comparable with the human syndrome, homozygous *Lmx1b* knockout mice lack patellae and suffer from severe podocyte damage. In contrast, however, podocin and the α3 and α4 chains of collagen IV are absent in the glomeruli of *Lmx1b* knockout mice. Further studies with podocyte-specific *Lmx1b* knockout mice have confirmed the importance of LMX1B in podocytes, as these mice apparently develop foot processes initially but lose them later on. We therefore conclude that LMX1B is essential for the development of metanephric precursor cells into podocytes and possibly also for maintaining the differentiation status of podocytes. LMX1B can serve as a model system to elucidate a genetic program in podocytes.

For many years, the mesangial cell took the forefront of glomerular research (see, for example, [1, 2]), but its pedestal was first slowly shaken by the painstaking morphological investigations of Wilhelm Kriz (confer [3, 4] for an early and late review, respectively) and finally abruptly toppled by irrefutable genetic evidence pointing towards the podocyte as a crucial cell in the glomerulus [5]. Meanwhile, the podocyte is firmly rooted in pathogenetic models of glomerular diseases and it is hard to imagine that it will leave again. Although mutations in *WT1*, a gene encoding a transcription factor of the Cys_2_His_2_-zinc finger family, had been found responsible for the podocytopathies Denys-Drash syndrome [6], WAGR syndrome [7] and Frasier syndrome [8], the article by Karl Tryggvason’s group on the identification of mutations in *NPHS1* added another dimension [5]. *NPHS1* is mutated in patients suffering from congenital nephrotic syndrome of the Finnish type. It codes for nephrin, a component of the slit diaphragm, and therefore is an essential part of the glomerular filtration barrier [9–11]. Since then several other genes have been cloned that when mutated lead to glomerular disease and which in the kidney are (almost) specifically expressed in podocytes. They are *LMX1B* [12–14], *NPHS2*, the gene encoding podocin [15]; *ACTN4*, the gene encoding α-actinin-4 [16]; *CD2AP* [17, 18] and *TRPC6* [19, 20]. In the following years, an increasing amount of evidence has accumulated on the specific role of these proteins in the podocyte. Nephrin and podocin participate in the formation of the slit diaphragm complex, α-actinin-4 crosslinks actin filaments in podocytes, and TRPC6 belongs to a special class of cation channels. Very little, however, is known about how the podocyte-specific expression of these genes is achieved. The sparse evidence that has been published concerns WT1, which binds to sequences in the promoter regions of the *Podxl* gene (encoding podocalyxin) [21] and of the *NPHS1*/*Nphs1*gene [22, 23]. Although WT1 activates the respective reporter constructs, the induction of the endogenous *NPHS1*/*Nphs1* gene by WT1 was described by one group [23] but not another [22].

Nail–patella syndrome has been known for many decades as a hereditary disease and was one of the first genetic disorders for which linkage was established. In addition to the obvious limb abnormalities, nephrologic symptoms develop in ∼40% of these patients over the course of several decades. On an ultrastructural level, the moth-eaten appearance of the glomerular basement membrane together with fibrillar deposits is considered typical of nail–patella syndrome. In addition, podocytes lose their foot processes (for references, see [24]). In 1998, not only were the first mutations in the *LMX1B* gene published for patients suffering from nail–patella syndrome [12–14], but a report also appeared on the first characterisation of the *Lmx1b* knock-out mouse [25]. A more careful analysis of the kidney phenotype in the *Lmx1b* knock-out mouse revealed pronounced retardation in the development of podocytes that did not elaborate foot processes and slit diaphragms. Consistent with this finding was the splitting of the glomerular basement membrane and the reduced number of endothelial fenestrations, because podocytes synthesise proteins of the glomerular basement membrane and control differentiation of glomerular endothelial cells [26, 27]. Attractive explanations for these morphological defects have come from the observations that the α3 and α4 chains of collagen IV are no longer detected in the glomerular basement membrane ([28] and personal observations), that the *Nphs2* gene is no longer expressed in podocytes of homozygous *Lmx1b* knock-out mice, and that podocytes in homozygous *Lmx1b* knock-out mice produce less vascular endothelial growth factor (VEGF) [26, 27]. Further molecular analysis demonstrated that LMX1B bound to AT-rich sequences in the first intron of the *COL4A4* gene [28] and in the promoter region of the *NPHS2* gene [26, 27].

Although the model that LMX1B activates the expression of *COL4A4* and *NPHS2*, and that inactivating mutations in the *LMX1B* gene secondarily lead to the loss of collagen IV and podocin and therefore to the characteristic alterations in nail–patella syndrome patients certainly is an attractive one, several caveats have to be mentioned as well. Firstly, we could not demonstrate activation of a reporter construct with 4.4 kbp of the *NPHS2* promoter by LMX1B [27], although another group showed an approximate twofold activation of the reporter gene controlled by four concatemerised LMX1B binding sites from the *NPHS2* promoter [26]. Secondly, when we stably transfected a human cervical carcinoma cell line HeLa cells (which admittedly bears only a very remote similarity to podocytes) with an LMX1B cDNA, we found no upregulation of the endogenous *NPHS2* gene [27]. Thirdly, podocin and the α3 and α4 chains of collagen IV were still present in glomeruli from patients with nail–patella syndrome [29]. And fourthly, the constitutive podocyte-specific inactivation of *Lmx1b* in the mouse does not lead to the loss of podocin or collagen IV [30]. What do these apparently discrepant results mean? If LMX1B already acts at a very early stage of podocyte development, specifically before the *NPHS2*, *COL4A3* and *COL4A4* genes are turned on (by other transcription factors?), the podocyte will just not have reached an advanced enough stage of development to produce podocin and collagen IV. In other words, LMX1B may rather exert a permissive influence and, for example, initiate the spreading of the foot processes upon which podocin would be produced and slit diaphragms be elaborated.

The constitutive podocyte-specific inactivation of *Lmx1b* represents a more comparable model for human nephropathy, but the mice only survive for ∼2 weeks after birth [30], again limiting their usefulness. In those animals, podocin and collagen IV α3/α4 are still present, and it appears as if foot processes and slit diaphragms are first elaborated and then lost secondarily (Fig. 1). Does LMX1B therefore play a role not only for the initial development of podocytes but also in the maintenance of their differentiation status? Clearly, more elaborate mouse models with an inducible inactivation of *Lmx1b* in adult animals are needed to answer this question. Such mice will also have the additional advantage of permitting the isolation of sufficient amounts of glomeruli, which can be used for DNA microarrays and identification of LMX1B target genes. Mouse genetics has already provided us with the verification of LDB1 as an interaction partner of LMX1B [30], and it may in the end help us to identify a genetic hierarchy acting in podocytes by telling us what factors control the expression of *LMX1B* in podocytes, what other proteins LMX1B interacts with and what genes are regulated by LMX1B in this peculiar cell type.

**Fig. 1:**
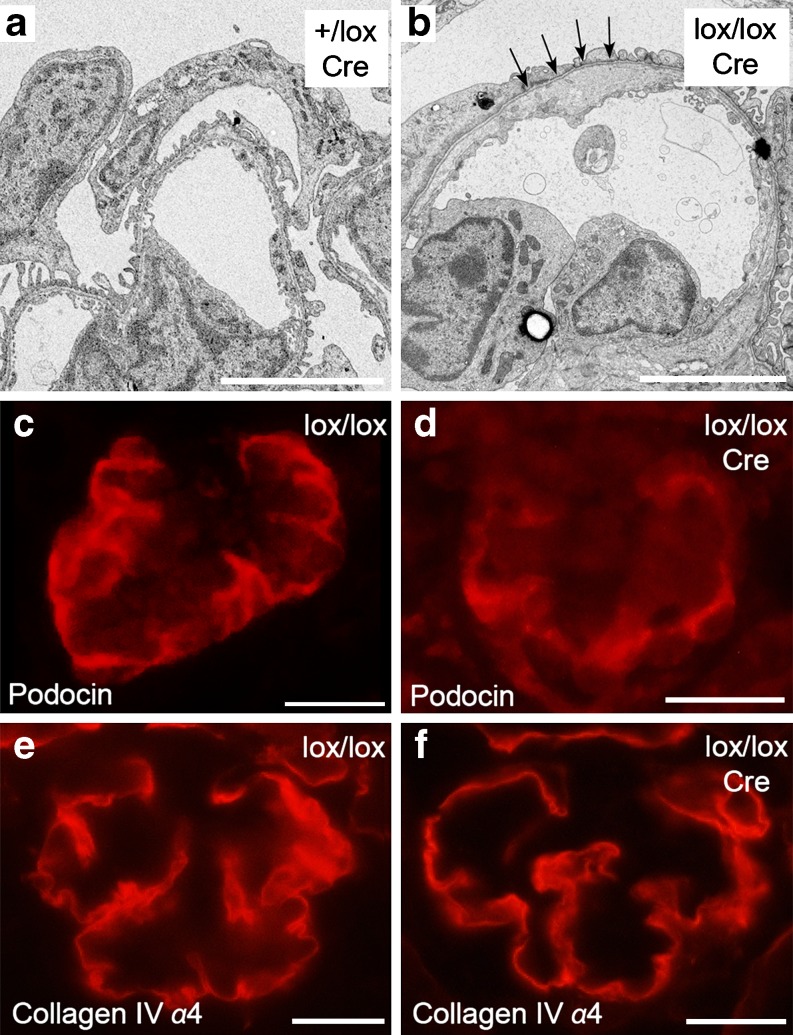
**a–f** Ultrastructural and immunohistochemical characterisation of mice with podocyte-specific inactivation of *Lmx1b*. In 11-day-old mice, the podocyte-specific inactivation of *Lmx1b* leads to the loss of foot processes and to a thickened glomerular basement membrane (*arrows* in** b**). However, despite the inactivation of *Lmx1b*, podocin and the α4 chain of collagen IV are still produced (**d**, **f**). *+/lox* control mice with one wild-type and one floxed *Lmx1b* allele; *lox/lox* mice with two floxed *Lmx1b* alleles; *Cre* presence of the Cre transgene under control of the human *NPHS2* promoter. *Bars*: 5 μm (**a**, **b**), 20 μm (**c**–**f**). With permission from [30]
